# Alzheimer’s disease brain contains tau fractions with differential prion-like activities

**DOI:** 10.1186/s40478-021-01127-4

**Published:** 2021-02-17

**Authors:** Longfei Li, Ruirui Shi, Jianlan Gu, Yunn Chyn Tung, Yan Zhou, Dingwei Zhou, Ruozhen Wu, Dandan Chu, Nana Jin, Kevin Deng, Jiawei Xu, Cheng-Xin Gong, Khalid Iqbal, Fei Liu

**Affiliations:** 1 Department of Neurochemistry, Inge Grundke-Iqbal Research Floor, New York State Institute for Basic Research in Developmental Disabilities, 1050 Forest Hill Road, Staten Island, NY 10314 USA; 2 Key Laboratory of Neuroregeneration of Jiangsu and Ministry of Education of China, Co-Innovation Center of Neuroregeneration, 19 Qixiu Road, Nantong, 226001 Jiangsu China

**Keywords:** Alzheimer’s disease, Tau pathology, Tau phosphorylation, Tau truncation, Prion-like seeding activity

## Abstract

Neurofibrillary tangles (NFTs) made of abnormally hyperphosphorylated tau are a hallmark of Alzheimer’s disease (AD) and related tauopathies. Regional distribution of NFTs is associated with the progression of the disease and has been proposed to be a result of prion-like propagation of misfolded tau. Tau in AD brain is heterogenous and presents in various forms. In the present study, we prepared different tau fractions by sedimentation combined with sarkosyl solubility from AD brains and analyzed their biochemical and pathological properties. We found that tau in oligomeric fraction (O-tau), sarkosyl-insoluble fractions 1 and 2 (SI_1_-tau and SI_2_-tau) and monomeric heat-stable fraction (HS-tau) showed differences in truncation, hyperphosphorylation, and resistance to proteinase K. O-tau, SI_1_-tau, and SI_2_-tau, but not HS-tau, were hyperphosphorylated at multiple sites and contained SDS- and β-mercaptoethanol–resistant high molecular weight aggregates, which lacked the N-terminal portion of tau. O-tau and SI_2_-tau displayed more truncation and less hyperphosphorylation than SI_1_-tau. Resistance to proteinase K was increased from O-tau to SI_1_-tau to SI_2_-tau. O-tau and SI_1_-tau, but not SI_2_-tau or HS-tau, captured tau from cell lysates and seeded tau aggregation in cultured cells. Heat treatment could not kill the prion-like activity of O-tau to capture normal tau. Hippocampal injection of O-tau into 18-month-old FVB mice induced significant tau aggregation in both ipsilateral and contralateral hippocampi, but SI_1_-tau only induced tau pathology in the ipsilateral hippocampus, and SI_2_-tau and HS-tau failed to induce any detectable tau aggregation. These findings suggest that O-tau and SI_1_-tau have prion-like activities and may serve as seeds to recruit tau and template tau to aggregate, resulting in the propagation of tau pathology. Heterogeneity of tau pathology within AD brain results in different fractions with different biological and prion-like properties, which may pose a major challenge in targeting tau for development of effective therapeutic treatments.

## Introduction

Alzheimer’s disease (AD) is characterized pathologically by extracellular amyloid β (A β) plaques and intracellular neurofibrillary tangles (NFTs) composed of abnormally hyperphosphorylated tau. Tau lesion (pretangles, neuropil threads, and NFTs), but not Aβ plaque load, is correlated with cognitive disturbances [2, 5, 23], suggesting a fundamental role of tau pathology in neurodegeneration of this disease.

In AD brain, tau pathology starts in the trans-entorhinal cortex, from where it spreads to limbic regions, followed by neocortical areas, according to the famous Braak stages [7, 8]. The distribution of NFTs associates with the progression of this disease [8, 23]. After examining the brains of younger cohorts and discovering NFTs in the locus ceruleus of a subset of individuals, Braak revised that subcortical nuclei may actually be the site of the initial seed for tau propagation [12]. Recently, tau tracer retention measured by positron emission tomography also showed similar stages [35, 51, 52]. Thus, tau pathology in AD brain may spread along neuroanatomical connections, which underlies the progression of AD.

The spatiotemporal spreading of tau pathology in AD was replicated recently in animal models. Clavaguera et al. injected brain extract from tau_P301S_ transgenic mice into the brain of wild-type tau-expressing mice and induced tau aggregation not only at the injection sites, but also in the anatomically connected brain regions in a time-dependent manner [13], leading to introduction of the concept of “propagation of tau pathology.” After this study, various mouse models were used to study the progressive propagation of tau pathology, including those using regional promoters, inoculation models, and viral models [1, 15, 16, 30, 32, 42, 47]. Tau pathology apparently radiates through the brain along synaptically connected pathways as the disease progresses.

As early as 1994, our group demonstrated that hyperphosphorylated cytosolic/oligomeric tau (AD p-tau) sequesters/captures normal tau in vitro to form filaments in a non-saturable manner [3], which was the first identification of prion-like activity of AD p-tau. Misfolded tau aggregates from brains of individuals with AD or tauopathies [6, 14, 30, 36] or from tau transgenic mouse brains [37] or generated in vitro [18, 27, 32, 50] are able to seed tau aggregation in cultured cells and in vivo. The seeding ability of tau from AD brains correlates positively with Braak stage and negatively with MMSE scores and precedes overt tau pathology [19]. In tau transgenic mice, tau seeds predict the spread of disease by appearing in brain regions prior to the appearance of any other pathological change [29]. Thus, the prion-like seeding activity of pathological tau may indicate the progression of tau pathology in AD.

**Fig. 1:**
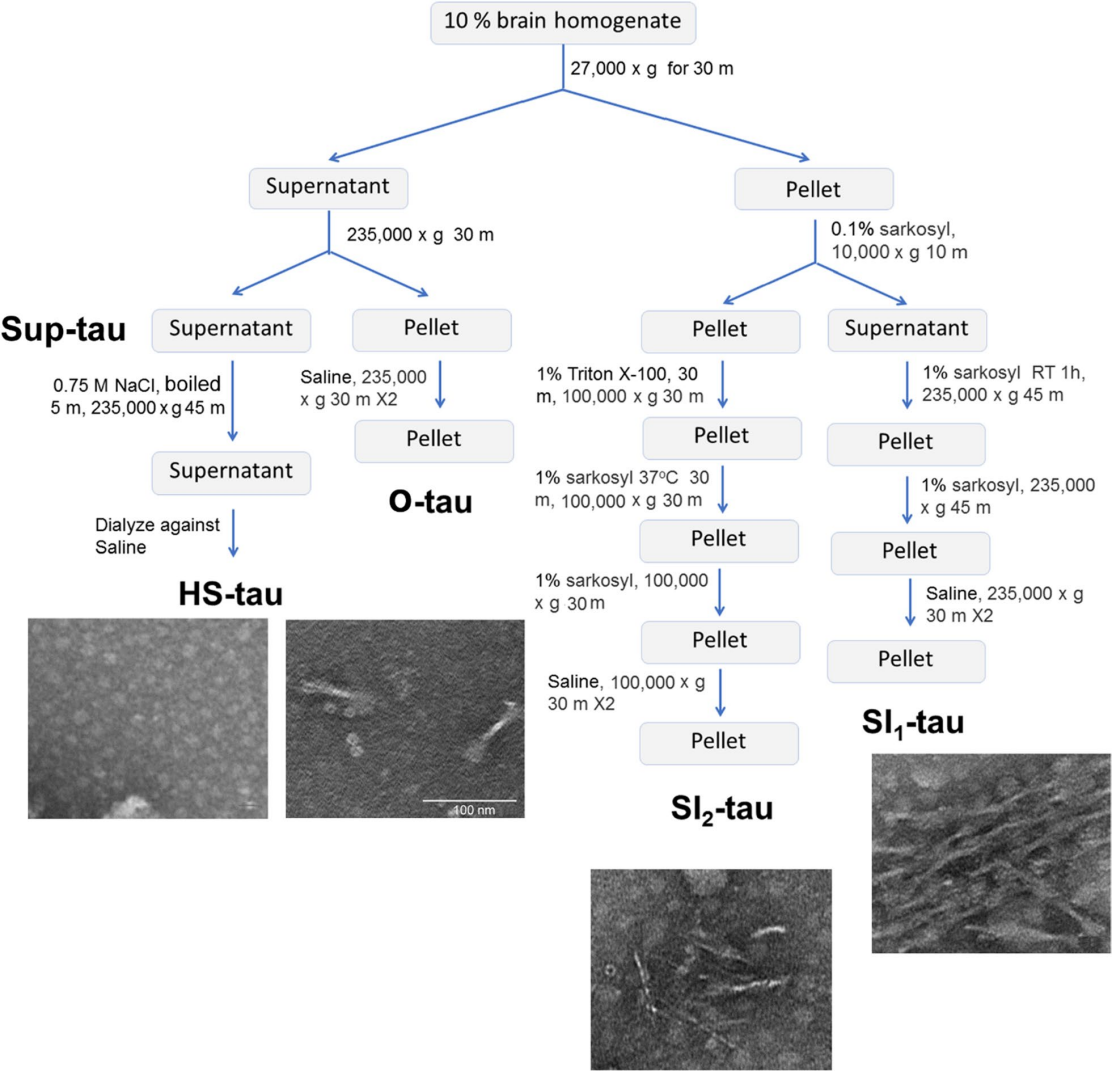
The main steps used for isolation of various tau fractions from AD brains and representative images of each fraction obtained by negative staining electron microscopy

Tau presents in different forms in AD brain [39], but its prion-like seeding activity is not well documented. By combining Kopke’s and Guo’s protocols [28, 39], we separated various tau fractions from AD brains, oligomeric fraction (O-tau), and sarkosyl-insoluble fractions 1 and 2 (SI_1_-tau and SI_2_-tau), and heat-stable tau (HS-tau) (Fig. 1), by sedimentation in sarkosyl buffer and assessed their seeding activity by using new methods we recently developed [26]. We found that different AD tau fractions displayed different properties in truncation, hyperphosphorylation, resistance to proteinase K, capturing normal tau in vitro and seeding tau aggregation in cultured cells and in vivo. O-tau and loose aggregates of tau in SI_1_-fraction showed prion-like activity, which is inert in compacted aggregates of tau in SI_2_-fraction and monomeric heat-stable tau.

## Materials and methods

### Isolation of various tau fractions from AD brain

Frozen brain tissue samples from autopsied and histopathologically confirmed AD cases with Braak Stages V and VI were obtained from the Brain Tissue Resource Center, McLean Hospital, Belmont, MA, USA. The use of autopsied frozen human brain tissue was in accordance with the National Institutes of Health guidelines and was exempted by the Institutional Review Board (IRB) of the New York State Institute for Basic Research in Developmental Disabilities because ‘‘the research does not involve intervention or interaction with the individuals’’ nor ‘‘is the information individually identifiable’’.

Tau fractions were isolated from autopsied and frozen AD cerebral cortex by a combination of the protocols described by Kopke and by Guo [28, 39]. Briefly (Fig. 1), 10% brain homogenate prepared in homogenization buffer (20 mM Tris–HCl, pH 8.0, 0.32 M sucrose, 10 mM β-mercaptoethanol (β-ME), 5 mM MgSO_4_, 1 mM EDTA, 10 mM glycerophosphate, 1 mM Na_3_VO_4_, 50 mM NaF, 2 mM benzamidine, 1 mM 4-(2-aminoethyl) benzenesulfonyl fluoride hydrochloride (AEBSF), and 10 μg/ml each of aprotinin, leupeptin, and pepstatin) was centrifuged at 27,000×*g* for 30 min. The pellet was saved for sarkosyl-insoluble tau (SI-tau) preparation. The supernatant was further centrifuged at 235,000×*g* for 30 min, and the resulting pellet, i.e., oligomeric tau–enriched fraction (O-tau), was collected and washed twice with saline and then resuspended in saline (Fig. 1). The supernatant, Sup-tau (Fig. 1), was used for HS-tau preparation.

Sarkosyl-insoluble aggregated tau preparation: The pellet from the 27,000×*g* centrifugation above was homogenized in the homogenization buffer containing 0.1% sarkosyl and centrifuged at 10,000×*g* for 10 min. The supernatant was adjusted to 1% sarkosyl, incubated for 1 h at room temperature (RT), and centrifuged at 235,000×*g* for 45 min. The pellet was washed once with 1% sarkosyl-homogenization buffer and washed twice with saline to obtain SI_1_-tau (Fig. 1). The pellet from the 10,000×*g* centrifugation above was incubated with 1% Triton X-100 in homogenization buffer for 30 min at RT and centrifuged for 1 h at 100,000×*g*. The resulting pellet was incubated in 1% sarkosyl in homogenization buffer for 1 h at RT and centrifuged at 100,000×*g* for 45 min. The resulting pellet was washed once with 1% sarkosyl in homogenization buffer and twice with saline and collected as SI_2_-tau (Fig. 1).

HS-tau preparation: The supernatant from the 235,000×*g* centrifugation above was adjusted to 0.75 M NaCl and 10 mM β-ME, heated for 5 min at 100 °C, and centrifuged at 235,000×*g* for 45 min. The resulting supernatant was dialyzed against saline; the tau in this pool was termed HS-tau (Fig. 1).

The tau preparations derived from AD brain described above were probe-sonicated for 5 min at 20% power and stored at − 80 °C until use.

### Negative staining electron microscopy

Various tau fractions were placed on 300 meshed carbon-coated copper grids for 1 min, stained with one drop of 2% Phosphotungstic acid for 1 min, and visualized with Hitachi HT7700 transmission electron microscope.

### Cell culture and transfection

HEK-293FT cells and HeLa cells were maintained in Dulbecco’s modified Eagle’s medium (DMEM) supplemented with 10% fetal bovine serum (FBS) (ThermoFisher Scientific, Waltham, MA, USA) at 37 °C (5% CO_2_). Transfections were performed with FuGENE HD (Promega, Madison, WI, USA) according to the manufacturer’s instructions.

### Western blots and immuno-dot blots

Samples were denatured by boiling in Laemmli buffer for 5 min. Protein concentration was measured using the Pierce™ 660 nm Protein Assay Kit (ThermoFisher Scientific). Samples were subjected to SDS-PAGE and transferred onto polyvinylidene fluoride membrane (Millipore Sigma, Burlington, MA, USA). The membrane was subsequently blocked with 5% fat-free milk-TBS (Tris-buffered saline) for 30 min, incubated with primary antibodies (Table 1) in 5% fat-free milk-TBS overnight, washed with TBST (TBS containing 0.05% Tween 20), incubated with peroxidase (HRP)-conjugated secondary antibodies (Jackson ImmunoResearch Laboratories, West Grove, PA, USA), washed with TBST, incubated with the ECL Western Blotting Substrate (ThermoFisher Scientific) and exposed to HyBlot CL® autoradiography film (Denville Scientific Inc., Holliston, MA, USA). Specific immunosignal was quantified by using the Multi Gauge software V3.0 from Fuji Film (Minato, Tokyo, Japan).

**Table 1:** Antibodies used in this study

Antibody	Type	Specificity	Species	Source/reference (cat/lot)
43D	Mono-	Pan-tau (a.a. 6–18)	M	In house/Biolegend (816601)
92e	Poly-	Pan-tau	R	In house [40]
R134d	Poly-	Pan-tau	R	In house [40]
111e	Poly-	Pan-tau	R	In house [40]
113e	Poly-	Tau (a.a. 19–32)	R	In house [40]
HT7	Mono-	tau (a.a. 159–163)	M	ThermoFisher (MN10000/LK152163)
Tau5	Mono-	tau (a.a. 210–230)	M	Millipore (MAB361/1816394)
77G7	Mono-	tau (a.a. 244–368)	M	In house/Biolegend (816701)
RD3	Mono-	3R-tau	M	Millipore (05-803/JBC1863429)
RD4	Mono-	4R-tau	M	Millipore (05-804/2073108)
QCB23070	Poly-	Up-tau (S46)	R	Gong et al. [21]
Tau-1	Mono-	Up-tau (S199/202)	M	Dr. Lester I. Binder
AT8	Mono-	p-tau (S202/T205)	M	ThermoScientific (MN1020/PI205175)
Anti-pT205	Poly-	p-tau (T205)	R	Invitrogen (44738G/RD214239)
Anti-pS214	Poly	p-tau (S214)	R	Invitrogen (44742G/0500B)
Anti-pT217	Poly-	p-tau (T217)	R	Invitrogen (44744/785771A)
AT180	Mono-	p-tau (T231)	M	Invitrogen (MN1040/SH2406086)
Anti-pS262	Poly-	p-tau (S262)	R	Invitrogen (44-750G/0204)
PHF1	Mono-	p-tau (S396/404)	M	Dr. Peter Davies
R145	Poly-	p-tau (S422)	R	In house [40]
Anti-GAPDH	Poly-	GAPDH	R	Sigma (G9545/015M4824V)
Anti-HA	Mono-	HA	M	Sigma (H9658/112M4841)
Anti-HA	Poly-	HA	R	Sigma (H6908/115M4872V)

*Mono*- monoclonal, *p*- phosphorylated, *up*- unphosphorylated, *Poly*- polyclonal, *M* mouse, *R* rabbit

Tau level in samples was assayed by immuno-dot blots as described previously. Briefly, various amounts of a sample were applied onto nitrocellulose (NC) membrane (Schleicher and Schuell, Keene, NH, USA) at 5 μl per grid of 7 × 7 mm in size. The blot was placed in a 37 °C oven for 1 h to allow the protein to bind to the membrane. Then, the membrane was processed as for Western blots described above by using a mixture of R134d and 92e pan tau antibodies as primary antibodies.

### Proteolysis of AD tau fractions by proteinase K

AD tau fractions (2.5 mg/ml) were incubated with various concentration of proteinase K in 10 mM Tris–HCl, pH 7.4, for 10 min at RT. The reaction was stopped by boiling in Laemmli buffer for 5 min. Proteolyzed tau was analyzed by Western blots.

### Tau capture/sequestration assay

Tau_151-391_ tagged with hemagglutinin (HA) was overexpressed in HEK-293FT cells. The cells, 48 h after transfection, were lysed in phosphate-buffered saline (PBS) containing 50 mM NaF, 1 mM Na_3_VO_4_, 1 mM AEBSF, 5 mM benzamidine, and 10 μg/ml each of aprotinin, leupeptin, and pepstatin by probe sonication at 20% power for 2 min. The cell lysates were centrifuged for 10 min at 10,000×*g*. The extract containing HA-tau_151-391_ was stored at − 80 °C until use.

Various amounts of AD tau fractions were dotted on nitrocellulose membrane and dried at 37 °C for 1 h. The membrane was blocked with 5% fat-free milk-TBS for 1 h and incubated with the above cell extract containing HA-tau_151-391_ overnight. After washing with TBST, captured HA-tau_151-391_ was detected by incubating with anti-HA in 5% milk-TBST and processed as described above for immuno-dot blots.

### AD tau fractions seed tau aggregation in cultured cells

HEK-293FT cells were transfected with pCI/HA-tau_151-391_ with FuGENE HD. Similar levels of tau in various AD tau fractions were mixed with Lipofectamine 2000 (3% in Opti-MEM) (ThermoFisher Scientific) in 50 μl for 20 min at RT and added to the cell cultures in 24-well plate after 6 h transfection. The cells were then lysed in RIPA buffer (50 mM Tris–HCl, pH 7.4, 150 mM NaCl, 1% NP-40, 0.5% sodium deoxycholate, and 0.1% SDS) containing 50 mM NaF, 1 mM Na_3_VO_4_, 1 mM AEBSF, 5 mM benzamidine, and 10 μg/ml each of aprotinin, leupeptin, and pepstatin for 20 min on ice after 42 h treatment. The cell lysates were centrifuged at 130,000×*g* for 45 min, and the resulting pellet was washed with RIPA buffer. The supernatants were pooled together as RIPA-soluble fraction and the pellet contained RIPA-insoluble fraction. Levels of RIPA-insoluble and -soluble tau were analyzed by Western blots developed with anti-HA.

To visualize tau aggregates induced by various AD tau fractions in cells, HA-tau_151-391_ was overexpressed in HeLa cells and treated with tau fractions for 42 h, as described above. The cells were then fixed for 15 min with 4% paraformaldehyde in phosphate buffer, washed with PBS, and treated with 0.3% Triton in PBS for 15 min at RT. After blocking with 5% newborn goat serum, 0.1% Triton X-100, and 0.05% Tween 20 in PBS for 30 min, the cells were incubated with anti-HA in blocking solution overnight at 4 °C, washed with PBS, and incubated with Alexa Fluor 488-conjugated-secondary antibody for 2 h at RT. After washing with PBS, the cells were mounted with ProLong™ Gold antifade reagent (ThermoFisher Scientific) and observed with a Nikon confocal microscope. The experiment was performed in triplicate wells and eight fields were photographed from each well of each group. Percentage of cells with aggregated tau was determined. Each experiment was repeated at least twice.

### Tau pathology templated by AD tau fractions in vivo

Similar amounts of tau in O-tau, SI_1_-tau, SI_2_-tau, and HS-tau determined by immuno-dot blots with mixture of two polyclonal pan-tau antibodies (92e and R134d) were injected into the hippocampus unilaterally in 18-month-old male and female FVB mice. Mice were deeply anesthetized and transcardially perfused with saline followed by buffered 4% paraformaldehyde 3 months after tau injection. Brain was post-fixed in the same fixation buffer overnight at 4 °C and dehydrated in buffered 30% sucrose solution. Brain was then cut into 40-μm serial coronal sections by using a freezing microtome, and the sections were collected in a 12-well plate containing antifreeze solution (30% glycerol and 30% ethylene glycol in 40% PBS) in sequence, and the free-floating sections were preserved in antifreeze solution at − 20 °C before immunohistochemical staining.

### Immunofluorescent staining

Brain sections from one well of a 12-well plate per mouse were washed with PBS and treated with 0.3% Triton in PBS for 15 min at RT. After blocking with 5% newborn goat serum, 0.1% Triton X-100, and 0.05% Tween 20 in PBS for 30 min, the sections were incubated with AT8 antibody in blocking solution overnight at 4 °C, washed with PBS, and incubated with Alexa 555-conjugated-second antibody for 2 h. Hoechst dye was used to stain nuclei. After washing with PBS, sections were mounted on microscope slides, air-dried, mounted with ProLong™ Gold antifade reagent (ThermoFisher Scientific), and set under a coverslip before imaging using a Leica TCS SP5 confocal microscope. For quantification, average AT8-positive neurons in contralateral and ipsilateral hippocampi were counted from three brain sections per mouse.

### Statistical analysis

The GraphPad Prism 6 software was used for statistical analysis. Results were analyzed by one-way ANOVA followed with Tukey's multiple comparisons test or by two-way ANOVA followed by Sidak’s multiple comparisons test for multiple-group analysis.

## Results

### AD tau fractions are truncated differentially

Tau in AD brain appears in various pools and in monomeric, oligomeric, and filamentous forms [39]. In addition to aggregated tau, AD brain also expresses similar levels of normal tau [38], which is heat-stable [17]. Aggregated tau is sarkosyl-insoluble [22]. Normal tau and pathological tau can be separated by sedimentation. In the present study, by combining Kopke’s and Guo’s protocols [28, 39], we isolated various tau fractions—O-tau, SI_1_-tau, SI_2_-tau, and HS tau—from AD brains (Fig. 1). Negative staining microscopy showed paired helical filaments in fraction SI_1_-tau, mostly straight filaments in SI_2_-tau, short filaments in O-tau, and non-filament in HS-tau (Fig. 1).

**Fig. 2:**
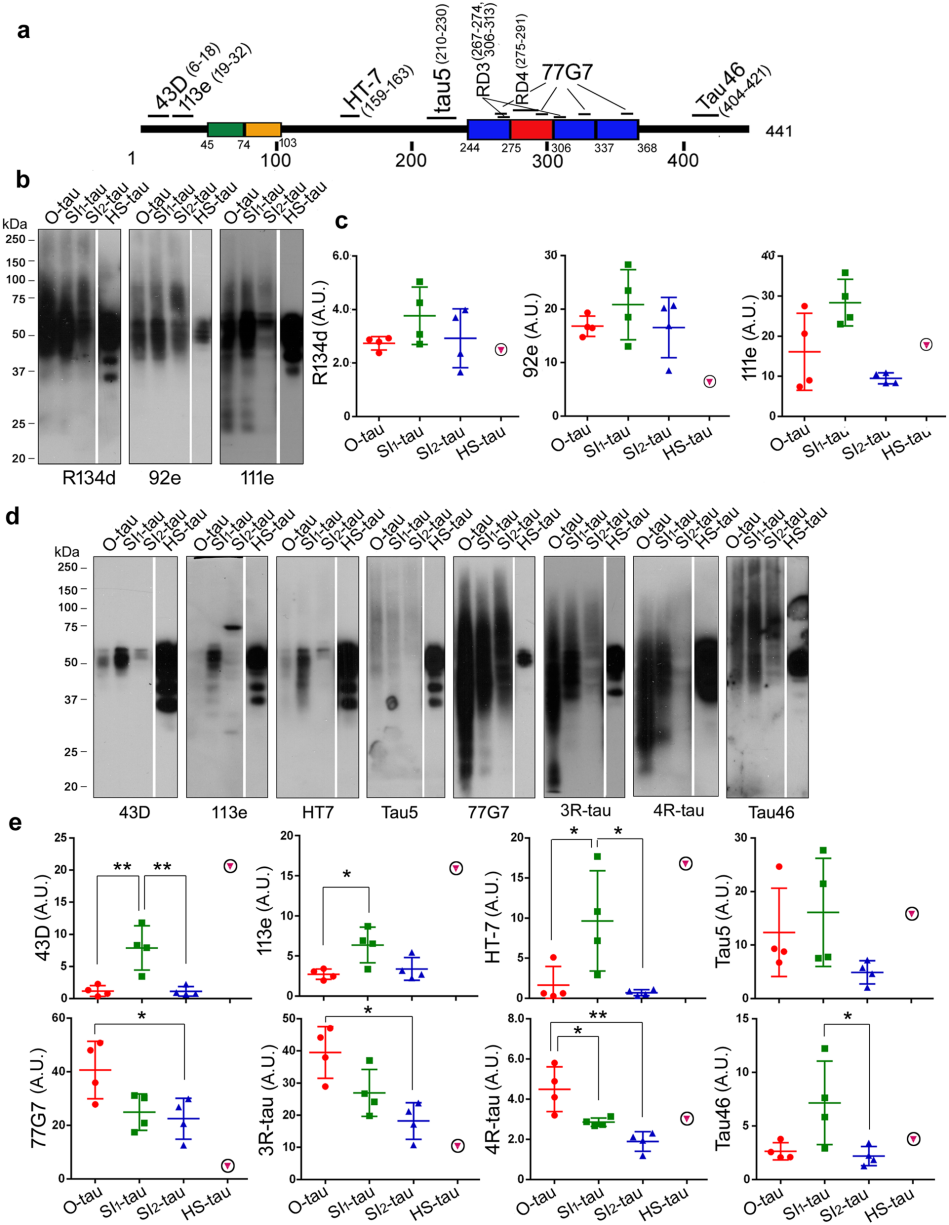
AD tau fractions are truncated at different sites. **a**. Schematic showing the position of epitopes of various tau antibodies used to analyze tau truncation. **b-e** Western blots of O-tau, SI_1_-tau, SI_2_-tau, and HS-tau from AD brains developed with polyclonal pan-tau antibodies (R134d, 111e, and 92e) (**b**), or with epitope-specific antibodies (**d**). The levels of tau in the four pools determined by the individual antibody were quantified densitometrically and are presented as mean $\pm$ SD (**c, e**). *p<0.05; **p<0.01. A.U., arbitrary units

It is widely believed that truncation of tau plays a critical role in tau pathogenesis [49, 54, 58]. To reveal the truncation of various AD tau fractions, we isolated aggregated O-tau, SI_1_-tau, and SI_2_-tau from four AD cerebral cortices and analyzed tau protein patterns by using polyclonal and monoclonal pan-tau antibodies to various regions of the protein (Fig. 2a). HS-tau from one AD brain was used as a reference. In general, we found SDS- and β-ME–resistant high-molecular weight-tau (HMW-tau) in O-tau, SI_1_-tau, and SI_2_-tau detected by R134d, 92e, and 111e (Fig. 2b). Heat treatment is known to remove aggregated tau [48]. We found no detectable HMW-tau in HS fraction (Fig. 2b). Compared with blots developed with antibodies R134d and 92e, a lesser amount of tau in SI_2_ fraction was detected with antibody 111e (Fig. 2b).

To determine tau truncations, O-tau, SI_1_-tau, SI_2_-tau, and HS-tau were analyzed by Western blots developed with a battery of tau antibodies targeting specific epitopes (Fig. 2a). Consistently, SDS- and β-ME–resistant HMW-tau was seen in O-tau, SI_1_-tau, and SI_2_-tau but not in HS-tau (Fig. 2d). HMW-tau was detected by Tau5, 77G7, RD3, RD4, and Tau46, but was not, or only weakly, stained by N-terminal antibodies 43D, 113e, and HT7 (Fig. 2d), suggesting that it is truncated at the N-terminus. Immunoactivities of the N-terminal antibodies 43D, 113e, and HT7 were less in O-tau and SI_2_-tau than in SI_1_-tau (Fig. 2d, e). Tau5 immunoactivity was slightly higher in O-tau and SI_1_-tau than in SI_2_-tau (Fig. 2d, e). Interestingly, immunoactivities of three antibodies against the microtubule-binding repeats, 77G7, RD3 and RD4, were similarly decreased from O-tau, SI_1_-tau to SI_2_-tau (Fig. 2d, e), but 77G7 revealed strong immunoactivity toward O-tau, SI_1_-tau, and SI_2_-tau (Fig. 2d, e). Compared with SI_1_-tau, a lower level of tau in O- and SI_2_-fractions was detected by Tau46 (Fig. 2d, e). Taken together, these results suggest that AD tau fractions are truncated differentially. O-tau and SI_2_-tau are truncated more at both N- and C-termini, compared with SI_1_-tau.

### AD tau fractions are hyperphosphorylated differentially

**Fig. 3:**
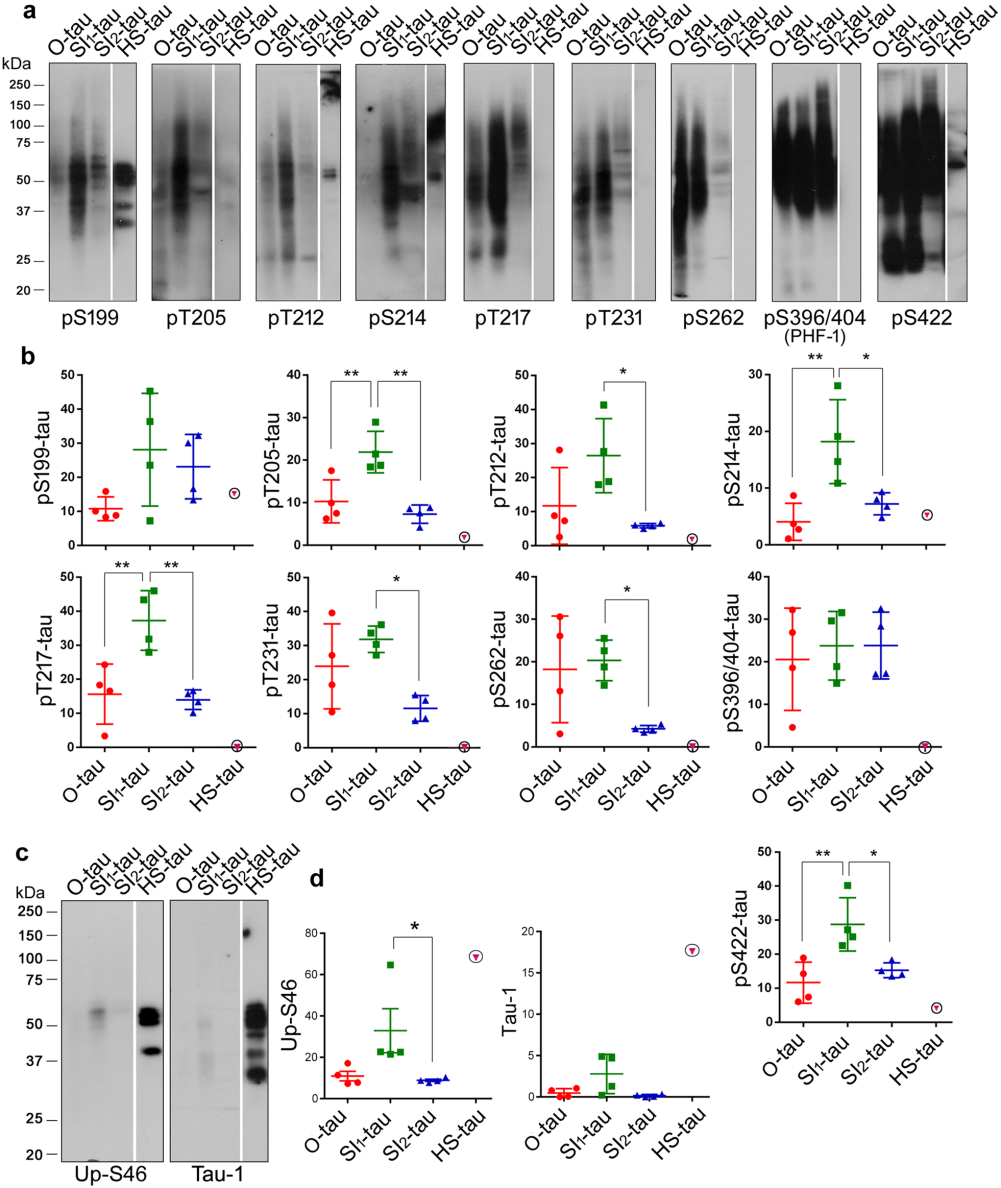
AD tau fractions are distinctively hyperphosphorylated. **a-d** AD tau fractions were analyzed by Western blots developed with site-specific and phosphorylation-dependent antibodies (**a, c**). The levels of hyperphosphorylated taus are shown as mean ± SD (**b, d**). HS-tau derived from an AD brain was included as a reference. *p<0.05; **p<0.01

Tau is hyperphosphorylated and aggregated into NFTs in AD and related tauopathies [24, 25, 33, 41]. To determine tau hyperphosphorylation, four AD tau fractions were subjected to Western blots developed with site-specific and phosphorylation-dependent tau antibodies. Consistently, we found HMW-tau in O-tau, SI_1_-tau, and SI_2_-tau, but not in HS-tau. HMW-tau was detected by all phospho-tau antibodies (Fig. 3a). O-tau, SI_1_-tau, and SI_2_-tau were hyperphosphorylated at Thr205, Thr212, Thr217, Ser231, Ser262, Ser396/404, and Ser422, but HS-tau was not, or only a little, phosphorylated at these sites (Fig. 3a, b). However, HS-tau was phosphorylated at Ser199 and Ser214 (Fig. 3a, b). Thus, O-, SI_1_-, and SI_2_-tau fractions, but not HS-tau, are hyperphosphorylated at multiple sites. The SDS- and β-ME–resistant HMW-tau is hyperphosphorylated.

O-tau and SI_2_-tau showed similar phosphorylation levels, but both were less phosphorylated than SI_1_-tau at Ser199, Thr205, Thr212, Ser214, Thr217, and Ser422 (Fig. 3a, b). O-tau and SI_1_-tau were phosphorylated similarly, but SI_2_-tau was less phosphorylated at Ser231 and Ser262, the sites in, or close to, microtubule-binding repeats (Fig. 3a, b). Similar phosphorylation levels of tau were observed in three AD tau fractions at Ser396/404 (Fig. 3a, b). Thus, these results suggest that O-tau, SI_1_-tau, and SI_2_-tau are hyperphosphorylated differentially. SI_1_-tau may be more hyperphosphorylated than O-tau and SI_2_-tau at most phosphorylation sites.

We also analyzed un-phosphorylated tau in these AD tau fractions by Western blots developed with antibodies to un-phosphorylated tau: Ser46 (Up-Ser46) and Tau-1 (Up-Ser195-202). We found no or very little un-phosphorylated tau at Ser46 and at Ser195-202 (Tau-1) in O-tau, SI_1_-tau, and SI_2_-tau, but marked levels in HS-tau (Fig. 3c, d). Moreover, SI_1_-tau contained more un-phosphorylated tau than O-tau and SI_2_-tau (Fig. 3c, d). These results showed that O-tau, SI_1_-tau, and SI_2_-tau are hyperphosphorylated, and HS-tau is less phosphorylated, at Ser46 and tau-1 sites. In addition to hyperphosphorylated tau, a small fraction of SI_1_-tau also is un-phosphorylated at Ser46 and tau-1 sites.

### AD tau fractions are resistant to proteinase K differentially

**Fig. 4:**
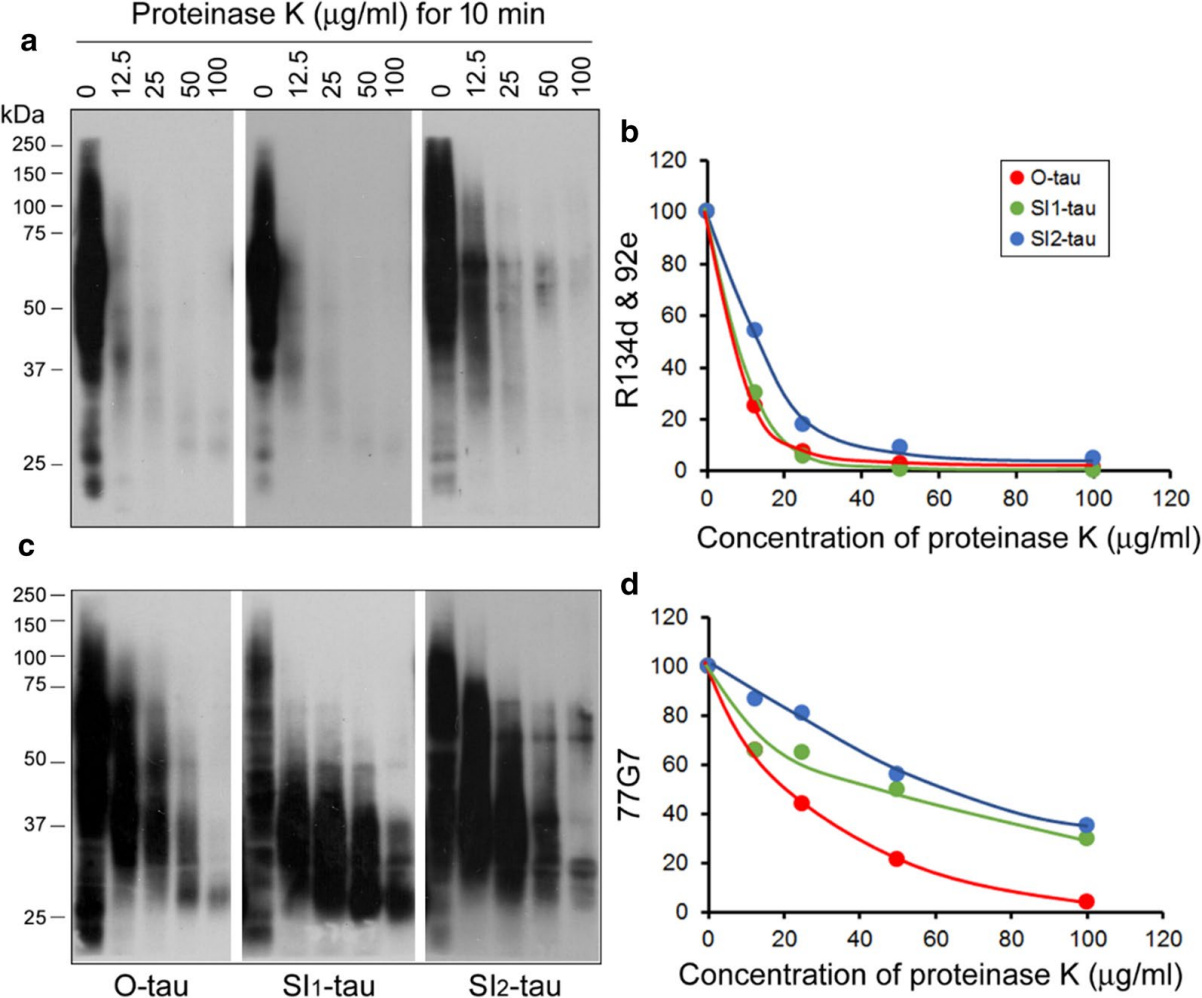
AD tau fractions are differentially resistant to proteinase K. AD tau fractions were incubated with various concentrations of proteinase K. The proteolyzed products were analyzed by Western blots developed with a mixture of R134d and 92e (**a**) and with 77G7 (**c**). The level of tau was quantified densitometrically and plotted against proteinase K concentration (**b, d**)

It is well known that tau in NFT is resistant to proteinase K [56]. To determine the sensitivity of these AD tau fractions to proteinase K, we incubated them with various concentrations of proteinase K for 10 min at room temperature and analyzed the digestion products by Western blots developed with 77G7 and with a mixture of R134d and 92e. We found that proteinase K proteolyzed O-tau, SI_1_-tau, and SI_2_-tau to small–molecular weight products that immunoreacted with 77G7 (Fig. 4c), but weakly with R134d/92e (Fig. 4a) in a dose-dependent manner. The greater reductions of tau by proteinase K were observed in the blots developed with R134d/92e than with 77G7 (Fig. 4b, d), suggesting that the microtubule-binding domain is relatively resistant to proteinase K. R134d/92e blot revealed that SI_2_-tau was decreased less rapidly than O-tau and SI_1_-tau (Fig. 4a, b), and 77G7 blots showed greater and faster reduction of O-tau than did SI_1_-tau and SI_2_-tau by proteinase K digestion (Fig. 4c, d). Both R134d/92e and 77G7 blots showed proteinase K–resistant 55- to 65-kDa tau in SI_2_-tau, but not in other fractions. Taken together, these results suggest that the amount of resistance to proteinase K was O-tau < SI_1_-tau < SI_2_-tau.

### AD tau fractions capture tau differentially

**Fig. 5:**
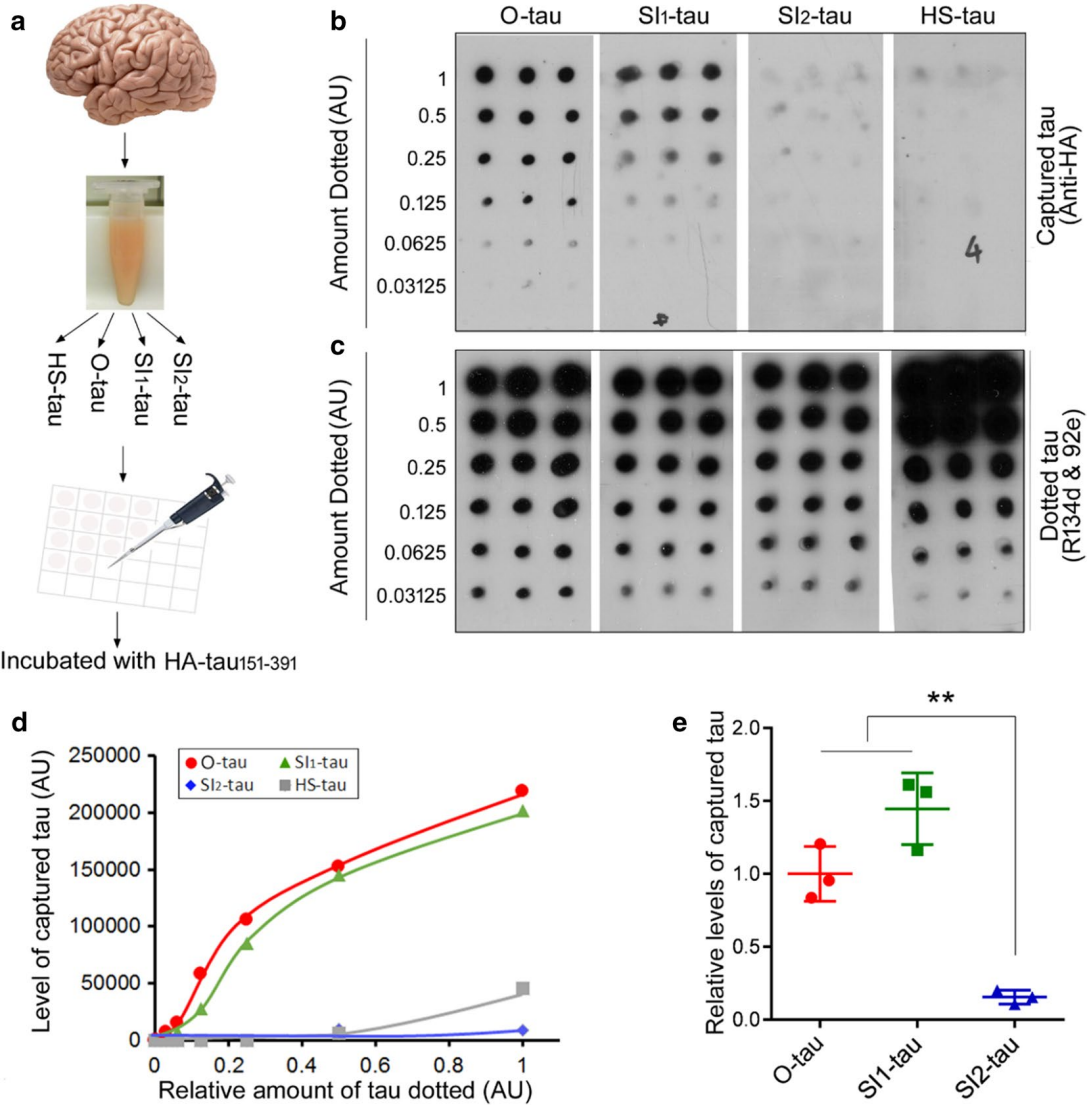
AD tau fractions capture tau differentially. **a** Schematic of tau capture assay. **b** Various mounts of AD tau fractions were dotted onto NC membrane. After blocking, the membranes were incubated with cell extract containing HA-tau_151-391_, and the captured tau was detected by anti-HA, followed by HRP-2^nd^ antibody and ECL. **c** Levels of tau in the fractions were analyzed by immuno-dot-blots developed with the mixture of R134d and 92e. **d** Levels of tau captured by the four tau fractions derived from the same AD cerebral cortex were plotted against the level of the dotted tau. **e** Levels of tau captured by O-tau, SI_1_-tau, and SI_2_-tau from three AD brains. The data are presented as mean ± SD. **p < 0.05

Aggregated and misfolded protein is able to recruit and to template the protein in normal conformation to the misfolded conformation, termed as the prion-like properties [20]. To determine the ability of AD tau fractions in recruiting tau, we performed overlay assay as reported recently [26]. We overexpressed tau_151-391_ tagged with HA in HEK-293FT cells. The crude extract of HEK-293/tau_151-391_ was used in tau capture assay (Fig. 5a). Tau_151-391_ comprises the β-sheet–forming core of the PHF structure. We dotted O-tau, SI_1_-tau, SI_2_-tau, and HS-tau with similar levels of tau on NC membrane. One membrane was incubated with the mixture of R134d and 92e to detect the levels of tau in the fractions. Another membrane was subjected to overlay assay. The membrane was incubated with HEK/tau_151-391_ extract after blocking. The captured tau was analyzed by anti-HA followed by HRP-2^nd^ antibody and ECL. The overlay assay revealed anti-HA immunoactivity in the membrane dotted with O-tau and SI_1_-tau, but not with SI_2_-tau and HS-tau, in a dose-dependent manner (Fig. 5b, d), indicating capture of tau_151-391_ by O-tau and SI_1_-tau, but not SI_2_-tau or HS-tau.

To further confirm capture of tau_151-391_ by O-tau and SI_1_-tau, but not by SI_2_-tau, three sets of O-tau, SI_1_-tau, and SI_2_-tau from three AD brains were prepared and subjected to the overlay assay (Fig. 5a). Again, we found that O-tau and SI_1_-tau were able, but SI_2_-tau was unable, to capture tau_151-391_ from the cell extract consistently (Fig. 5e).

### AD tau fractions seed tau aggregation in cultured cells differentially

**Fig. 6:**
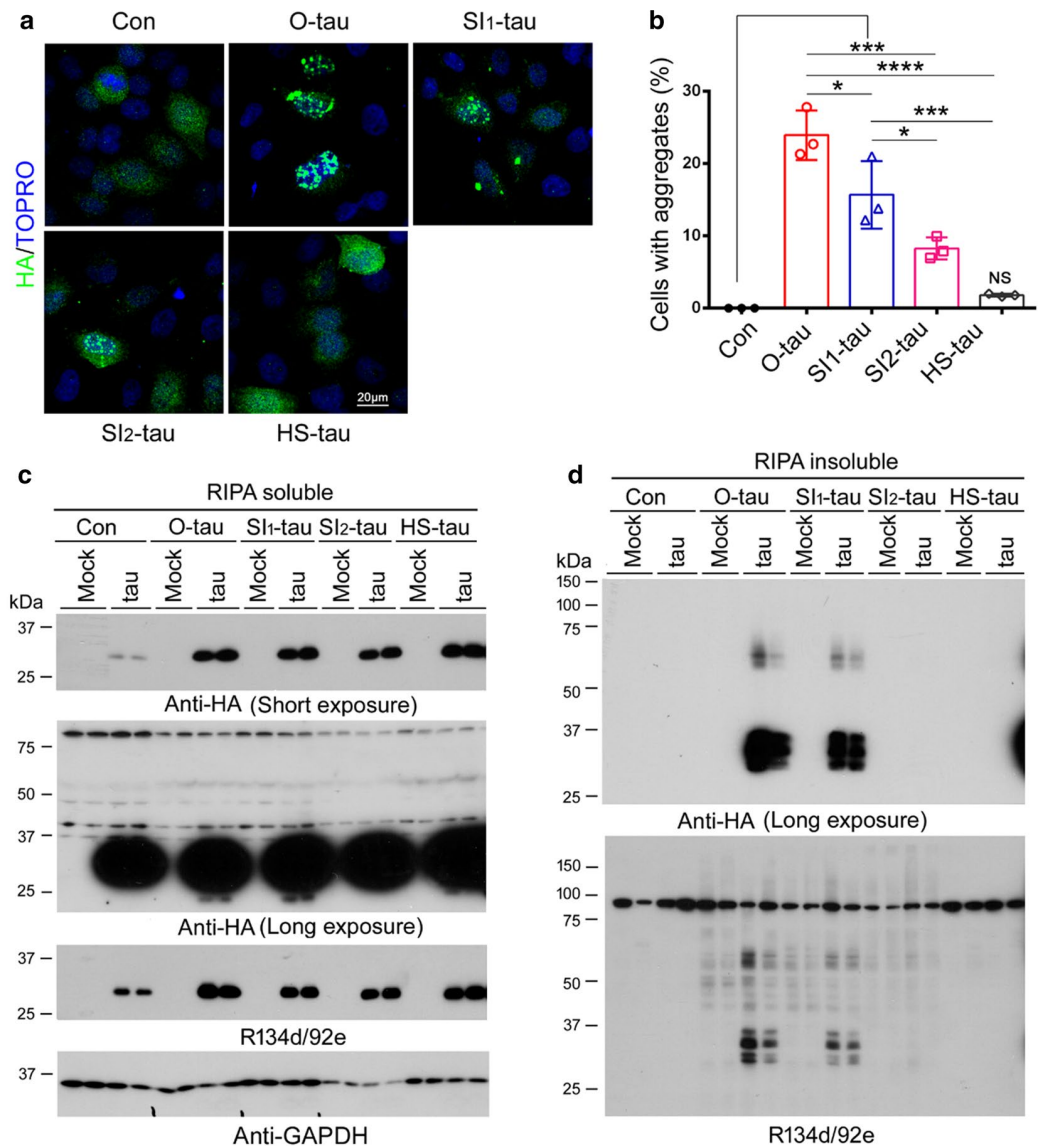
AD tau fractions template tau aggregation in cultured cells distinctively. **a, b** HeLa cells were transfected with pCI/HA-tau_151-391_ and treated with AD tau fractions containing similar tau levels. The cells were immunostained with anti-HA, followed by Alex488-Anti-mouse IgG. The numbers of cells with tau aggregation were counted. The data on the percentage of cells with aggregation are presented as mean ± SD. *p < 0.05, ***p < 0.001, ****p < 0.0001. **c, d** HEK-293FT/HA-tau_151-391_ cells treated with AD tau fractions containing similar tau levels. The cells were lysed in RIPA buffer 42 h after treatment. RIPA-soluble (**c**) and -insoluble (**d**) taus were analyzed by Western blots developed with anti-HA and a mixture of R134d and 92e

Seeding tau aggregation by misfolded tau in cultured cells is the basis of prion-like activity. We recently reported deletion of the first 150 and the last 50 amino acid (a.a.) of tau enhanced its aggregation seeded by AD O-tau [26]. To determine the seeding activity of these AD tau fractions, we overexpressed HA-tau_151-391_ in HeLa cells and treated the cells with O-tau, SI_1_-tau, SI_2_-tau, or HS-tau containing similar tau levels for 42 h after 6 h transfection. The cells were immuno-stained with anti-HA, and the numbers of cells with aggregates were counted. No aggregated HA-tau_151-391_ was observed in the cells without treatment with AD tau fractions (Fig. 6a, b). O-tau treatment induced ~ 25% cells with tau aggregates, and SI_1_-tau induced ~ 15% cells with aggregation. Significantly fewer cells were induced to form aggregates by SI_2_-tau, and no significant tau aggregates were formed by HS-tau (Fig. 6a, b). Thus, these results suggest the strongest seeding activity of O-tau. The seeding activity of AD tau fractions was reduced gradually from O-tau, SI_1_-tau, SI_2_-tau, to HS-tau.

To analyze biochemically the tau aggregation induced by the AD tau fractions, we also lysed the cells with RIPA buffer and analyzed the levels of tau in RIPA-soluble and -insoluble fractions by Western blots developed with anti-HA and a mixture of R134d and 92e. Anti-HA blots revealed no soluble and insoluble tau in the mock cells treated with AD tau fractions (Fig. 6c, d). The levels of RIPA-soluble tau_151-391_ were found to be increased in cells treated with the tau fractions (Fig. 6c). A significant amount of RIPA-insoluble tau was found in HEK-293FT/HA-tau_151-391_ cells treated with O-tau and SI_1_-tau, but not in cells treated with SI_2_-tau or HS-tau (Fig. 6d). We found only one 28-kDa band in RIPA-soluble fraction, but three major bands in RIPA-insoluble fraction in cells treated with O-tau and SI_1_-tau, which suggested hyperphosphorylation of RIPA-insoluble tau. In addition to ~ 28- to 32-kDa tau, we found 60- to 65-kDa SDS- and β-ME–resistant HMW-tau in RIPA-insoluble factions in O-tau– and SI_1_-tau–treated cells (Fig. 6d), but not in corresponding RIPA-soluble factions (Fig. 6c). R134d/92e blots showed immunoactivity in tau strain–treated mock cells, but clearly more tau in HEK-293FT/HA-tau_151-391_ cells treated with O-tau and SI_1_-tau, compared with control treatment (Fig. 6d). Taken together, these results suggest that O-tau and SI_1_-tau, but not SI_2_-tau and HS-tau, have prion-like properties to seed tau aggregation in cultured cells.

### Heat treatment does not passivate the prion-like activity of pathological tau

**Fig. 7:**
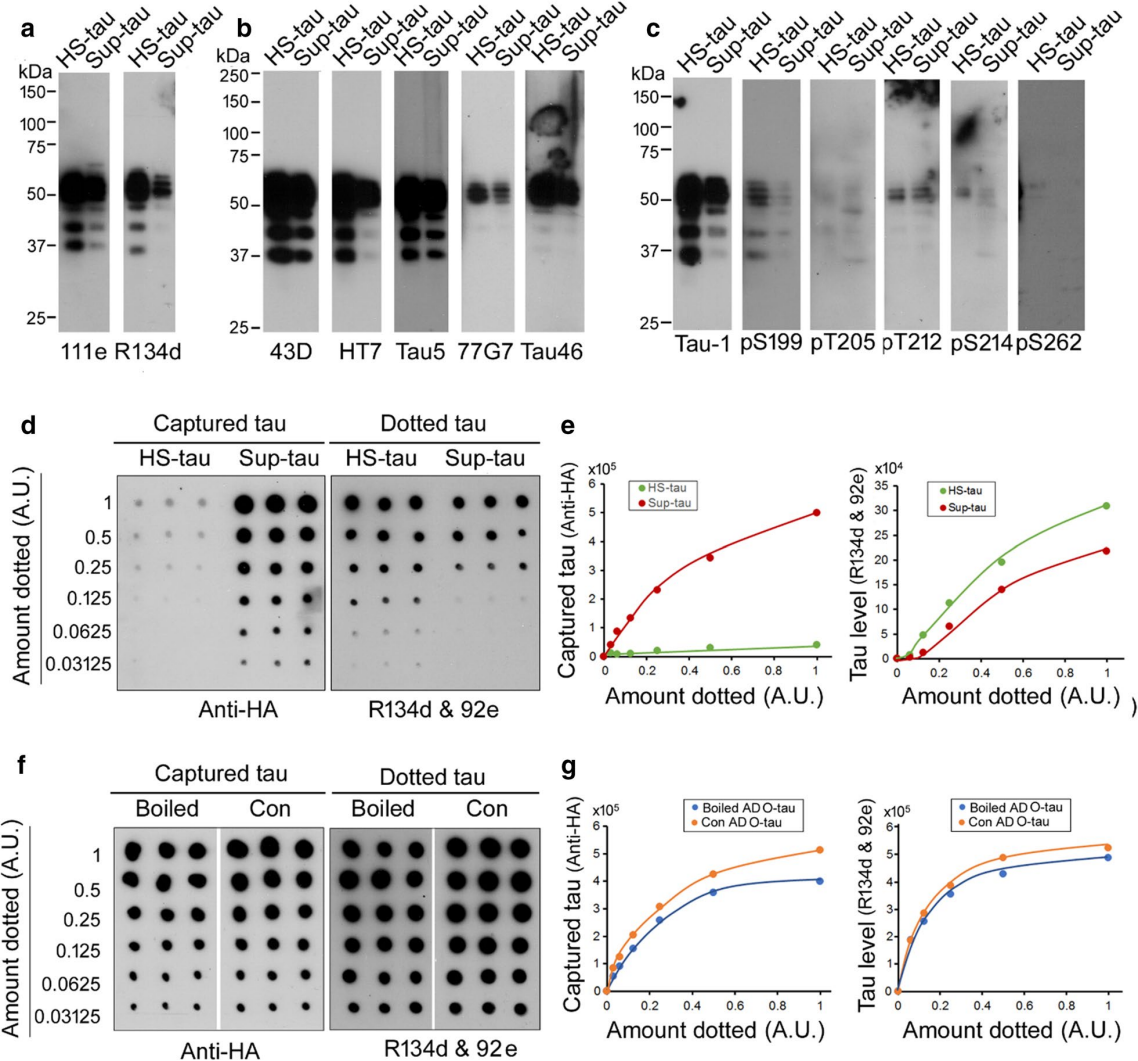
Heat treatment does not passivate the prion-like activity of O-tau. **a-c** Sup-tau and HS-tau were similar in phosphorylation and truncation. Sup-tau and HS-tau were analyzed by Western blots developed with polyclonal tau antibodies (**a**), monoclonal tau antibodies (**b**), and site-specific and phosphorylation-dependent antibodies (**c**). **d, e** Sup-tau, but not HS-tau, was able to capture tau. Various amount of Sup-tau and HS-tau were applied on NC membranes. One membrane was subjected to tau overlay assay by sequential incubation of HEK-293FT/HA-tau_151-391_ cell extract, anti-HA (**d**, left), and one membrane was subjected to analysis of tau level by dot-blot developed with a mixture of R134d and 92e (**d**, right). The captured tau (**e**, left) and the dotted tau levels (**e**, right) detected in panel d were plotted against applied Sup-tau and HS-tau. **f, g** Heat treatment did not affect O-tau to recruit tau. Various amounts of O-tau were applied onto NC membrane. One membrane was boiled, and the other membrane was treated with the same buffer at RT as a control. The membranes were incubated with cell extract containing HA-tau_151-391_. The captured tau (**f**, left) and applied tau (**f**, right) were analyzed as described above and plotted with the levels of O-tau (**g**). A.U., arbitrary units

HS-tau derived from 235,000×*g* supernatant (Sup-tau) of brain homogenate by heat treatment (Fig. 1) did not show prion-like activities (Figs. 5, 6). HS-tau was less truncated and least phosphorylated. We found that both Sup-tau and HS-tau were almost similar in Western blots developed with polyclonal pan-tau antibodies (Fig. 7a), monoclonal antibodies against specific epitopes (Fig. 7b), and site-specific and phosphorylation-dependent tau antibodies (Fig. 7c), suggesting that heat treatment did not affect the truncation and phosphorylation of tau.

Next, we performed overlay assay as described above to compare the ability of Sup-tau with HS-tau to recruit tau. We found that consistently HS-tau could not capture tau, but Sup-tau was able to recruit tau from HEK-293FT/tau_151-391_ cell extract (Fig. 7d, e), when even a smaller amount of tau in Sup-fraction was applied (Fig. 7d, e). These results suggest that either heat treatment inactivates tau’s ability to bind to tau_151-391_ or the Sup-tau might contain the small-size oligomers. It is well known that heat treatment removes aggregated tau [48]. Thus, unlike HS-tau, Sup-tau might contain small-size oligomeric tau.

We then studied whether heat treatment kills the prion-like activity of pathological tau. We dotted various amounts of O-tau on NC-membranes parallelly. One set of membranes was boiled in 50 mM Tris–HCl, 0.75 M NaCl, for 10 min to mimic heat treatment as the HS-tau preparation. Another membrane was incubated with the same buffer at RT as control treatment. Then, the membranes were overlaid with the HEK-293FT/tau_151-391_ cell extract for tau capture assay or with a mixture of R134d and 92e for tau assay, as described above. We found that heat treatment caused a slight reduction of tau level (Fig. 7f, g). Similarly, a slightly lesser amount of tau was captured by heat-treated O-tau than by control O-tau (Fig. 7f, g), suggesting that the heat treatment did not affect the prion-like activity of O-tau.

### AD tau fractions induce tau pathology in vivo differentially

**Fig. 8:**
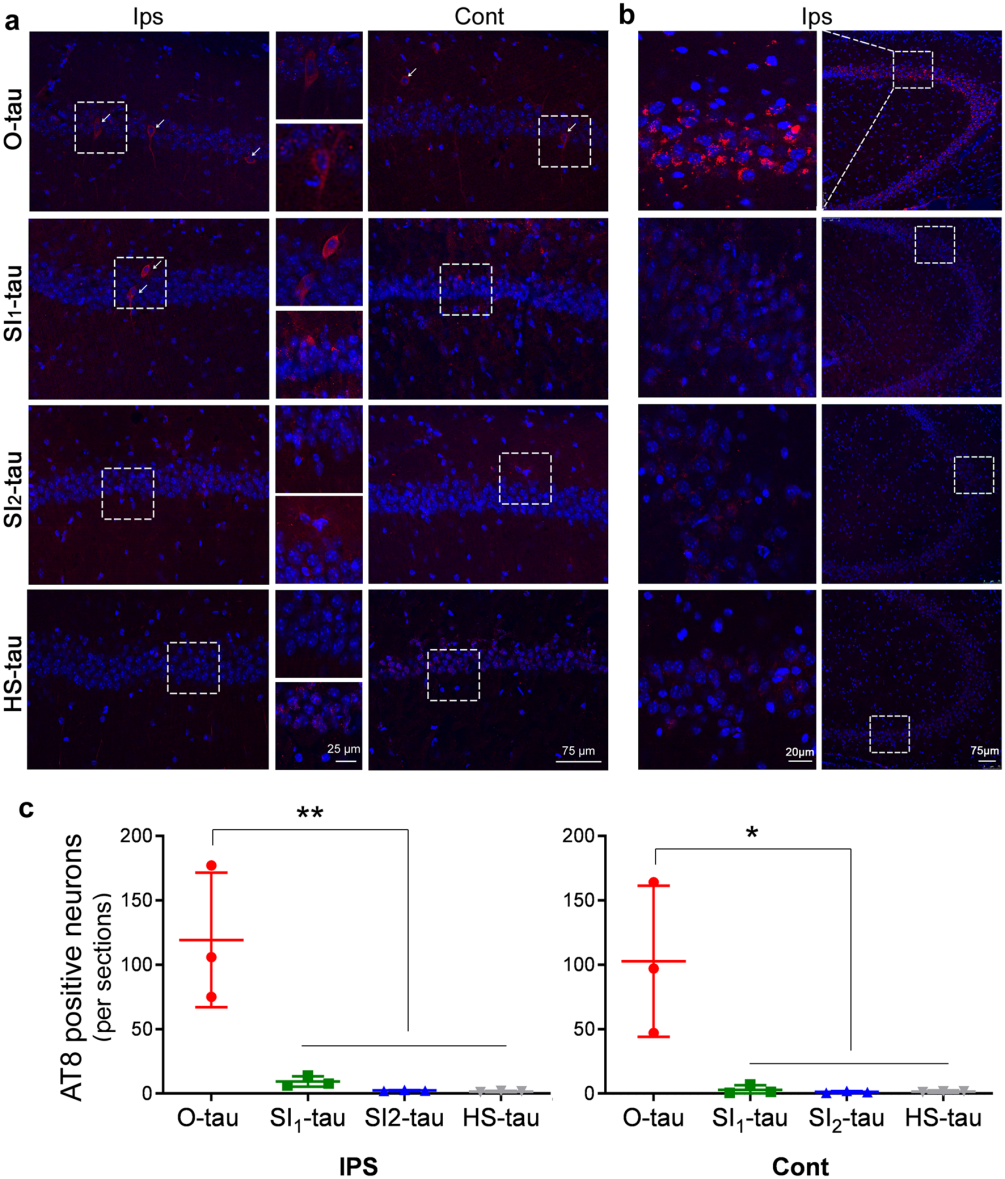
AD tau fractions induce tau pathology differentially in vivo. **a, b** AD tau fractions at similar tau levels were injected into hippocampus of 18-month-old-FVB mice. Brain sections were immunostained with AT8 3 months after injection. Hoechst dye was used to stain nuclei. Representative AT8 immunostaining of ipsilateral (Ips) and contralateral (Cont) CA1s (**a**) and Ips CA3 (**b**) of the mouse hippocampus after injection with AD tau fractions. **c** AT8-positive neurons in Ips hippocampus (left) and Cont hippocampus (right) were quantified. Data are presented as mean ± SD, **P<0.05; **P<0.01

To determine the seeding activity of AD tau fractions in vivo, we injected the four AD tau fractions with similar tau levels into the hippocampus in 18-month-old FVB mice and analyzed tau pathology in ipsilateral and contralateral hippocampi by immunofluorescence staining with AT8 3 months after injection. We found many AT8-positive neurons in both the ipsilateral and contralateral CA1 (Fig. 8a) and the ipsilateral CA3 (Fig. 8b) of the hippocampi of mice injected with O-tau, and a few AT8-positive neurons in the ipsilateral CA1 (Fig. 8a) of SI_1_-tau–injected mice. No obvious AT8 immunoactivity was observed in the ipsilateral and contralateral hippocampi of SI_2_-tau– or HS-tau–injected mice. AT8 immunostaining was not detectable in the cortex of the mice injected with any of the four tau fractions. AT8 immunostaining was dramatically higher in both the ipsilateral and contralateral hippocampi injected with O-tau than in those injected with SI_1_-tau, SI_2_-tau, or HS-tau (Fig. 8c), suggesting that O-tau serves as potent seeds to induce tau pathology in vivo.

## Discussion

Tau in AD brain exists in monomeric, oligomeric, and fibric forms. In the present study, we isolated from AD brain four fractions—O-tau, SI_1_-tau, SI_2_-tau, and HS-tau—and analyzed their biochemical and prion-like properties. We found that O-tau, SI_1_-tau, and SI_2_-tau, but not HS-tau, contained SDS- and reducing agent–resistant HMW-tau. O-tau and SI_2_-tau revealed similar patterns of truncation and hyperphosphorylation. Compared to O-tau and SI_1_-tau, SI_2_-tau was more resistant to proteolysis by proteinase K. O-tau, SI_1_-tau, but not SI_2_-tau or HS-tau, captured/sequestered tau in vitro and templated tau aggregation in cultured cells. Heat treatment did not inactivate the prion-like activity of O-tau. O-tau induced tau pathology in the ipsilateral and contralateral hippocampi, SI_1_-tau only induced it in the ipsilateral hippocampus, and SI_2_-tau or HS-tau could not induce tau aggregation in the hippocampi of 18-month-old FVB wild-type mice, as determined 3 months after injection. These results suggest that tau in the four isolated fractions showed distinct biochemical and prion-like properties. O-tau and SI_2_-tau showed similarities in hyperphosphorylation and truncation. Oligomeric O-tau and SI_1_-tau in AD brain may serve as seeds to induce tau aggregation. Monomeric tau and SI_2_-tau were inert in the prion-like properties, and they could not induce tau pathogenesis.

Tau is truncated at multiple sites in AD brain [49, 54]. Truncation may facilitate tau pathogenesis. We found in a parallel study that deletion of the first 150 or 230 a.a. and the last 50 a.a. enhanced tau’s site-specific hyperphosphorylation and self-aggregation as well as its binding to, and its aggregation seeded by, O-tau. tau_151-391,_ corresponding to the β-sheet–forming core of the PHF structure [46, 55], contains microtubule-binding repeats and showed the highest pathology-associated activities. The microtubule-binding repeats of tau (tauRD) with P301S mutation have been used in HEK293-tau-biosensor cells for tau-seeding [57]. Tau_151-391_ aggregates induced by O-tau were thioflavin T–positive and showed SDS- and β-ME–resistant HMW-tau in Western blots. By using RIPA buffer, aggregated tau was yielded in RIPA-insoluble fraction.

O-tau yielded from 27,000×*g* to 235,000×*g* fraction of AD brain homogenate [39]. Different from the tau in the 235,000×*g* supernatant, O-tau was abnormally hyperphosphorylated and formed SDS- and β-ME–resistant HMW aggregates, which lacked the N-terminal portion. O-tau displayed very potent prion-like activities, capturing/sequestering tau and seeding tau aggregation in cultured cells and in vivo. Heat treatment removes aggregated tau from the supernatant [48]. We found that heat treatment did not change O-tau ability to capture tau. However, HS-tau derived from Sup-tau could not capture tau, suggesting that aggregated tau, but not monomeric tau has prion-like activity. It was reported that tau trimers are the minimal propagation unit spontaneously internalized to seed intracellular aggregation [44]. However, large (> 10 mer) aggregated tau, but not small, oligomeric (< 6 mer) tau, from P301S transgenic mouse brains seeded cellular tau aggregation [34]. Similarly, we previously found that compared with O-tau from AD brain, O-tau from 3xTg-AD mouse brain showed much weaker seeding activity [40]. Furthermore, tau monomer purified from AD brain also had intrinsic seeding activity, and self-associated to produce larger seed-competent assemblies. It was proposed that tau monomer occupies two distinct and stable conformational ensembles: inert and seeding-competent [45]. Thus, we speculate that the 235,000×*g* supernatant contains small aggregates or/and seeding-competent tau monomer that may not be SDS- and β-ME–resistant but could capture tau and heat-treatment removes both species of tau.

Similarly, serial sedimentation can divide tau from AD brain into various fractions [53]. It was found that 3,000×*g* and 10,000×*g* AD brain extracts, which presumably contained HMW proteins, could be up-taken by cultured neurons, but 50,000×*g* and 150,000×*g* extracts, from which HMW tau was depleted by sedimentation, could not be up-taken by neurons [53]. The 3,000×*g* brain extracts showed significantly higher seeding activity than 150,000×*g* extracts. Thus, the 3,000×*g* and 10,000×*g* extracts contain various sizes of oligomeric tau [53], which may serve as predominant seeds to template tau aggregation. Consistently, we found here that O-tau from 27,000×*g* to 235,000×*g* displayed the prion-like activities.

A previous study showed that AD tau from the 10,000×*g* brain homogenate in 0.1% sarkosyl-high salt buffer to 235,000×*g* in 1% sarkosyl induced tau aggregation in vitro and in vivo [28]. Different from this protocol, we first separated monomeric and oligomeric tau from aggregated tau by centrifugation of brain homogenate at 27,000×*g* and then incubated the pellet in the buffer containing 0.1% sarkosyl and centrifuged at 10,000×*g*. The supernatant and the pellet probably contain loose and compressed tau aggregates, respectively. These fractions were incubated in 1% sarkosyl containing buffer and centrifuged at 100,000×*g* to yield SI_1_-tau and SI_2_-tau. Both SI_1_ and SI_2_ fractions contained SDS- and β-ME–resistant HMW-tau, which did not react with N-terminal tau antibodies and was hyperphosphorylated at multiple sites. A relatively higher level of tau was detected in the SI_1_ fraction by the N-terminal antibodies than in the SI_2_ fraction. Compared to O-tau, less tau was detected by the antibodies against microtubule-binding repeats, suggesting that SI_1_-tau and SI_2_-tau may be less accessible to these antibodies. Misfolded protein aggregates are usually resistant to proteolysis. We found that the resistance of O-tau, SI_1_-tau, and SI_2_-tau to proteinase K digestion was increased. Most interestingly, SI_1_-tau was able to capture tau and to seed tau aggregation in cultured cells in prion-like fashion, but SI_2_-tau was inert in these prion-like properties.

Prion-like spread of misfolded tau aggregates might underlie the stereotypic progression of neurodegenerative tauopathies. We reported in 1994 that the cytosolic and hyperphosphorylated tau from AD brain, named AD p-tau, sequestered tau and induced tau aggregation in vitro, which is the first study showing the prion-like activity of AD p-tau [3, 4]. AD p-tau was further purified by ion-exchange chromatography to remove non-hyperphosphorylated tau in O-tau [39]. Different from AD p-tau, PHF-tau could not sequester normal tau [4]. In the present study, we found that O-tau and SI_1_-tau, but not SI_2_-tau, captured tau and templated tau aggregation. We speculate that the major component of SI_2_-tau may be PHF-tau.

Tau pathology initiates in the subcortical regions, transentorhinal cortex, and entorhinal cortex (stages I and II), then appears in the hippocampal formation and some parts of the neocortex (stages III and IV), followed by most of the neocortex (stages V and VI) [7, 10]. Immunohistochemical (IHC) study with AT8, a principal tool to define AD intraneuronal pathology [43], showed that AT8 signal first appears in the locus coeruleus (LC), suggesting that tau aggregation in the LC may represent the earliest phase of AD pathogenesis [9, 11]. Individuals at stages I and II are asymptomatic, but over half of the subjects at NFT stages III–IV exhibited signs of mild cognitive impairment, and over 90% of subjects at NFT stages V–VI showed moderate to severe dementia [31]. By using HEK293-tau-biosensor cells, tau-seeding activity from AD brains correlates positively with Braak stage and negatively with MMSE [19], but pathological seeding activity begins in the transentorhinal/entorhinal cortices (TRE/EC) rather than in the LC [36]. In the present study, we analyzed various tau fractions isolated from AD cerebral cortex at NFT stages V-VI and found that various AD tau fractions displayed different biochemical and prion-like properties, indicating the heterogenicity of pathological activity of tau fractions in AD brains.

In addition to AD, aggregated tau is a common feature of tauopathies. Distinct tau fractions from various tauopathies have been shown to induce distinct tau aggregation in mouse brains [50]. Thus, heterogeneity of tau pathology both within AD and among different tauopathies could be due to different fractions with different biological and prion-like properties. This heterogeneity poses a major challenge in targeting tau for development of effective therapeutic treatment for tauopathies.

**Fig. 9:**
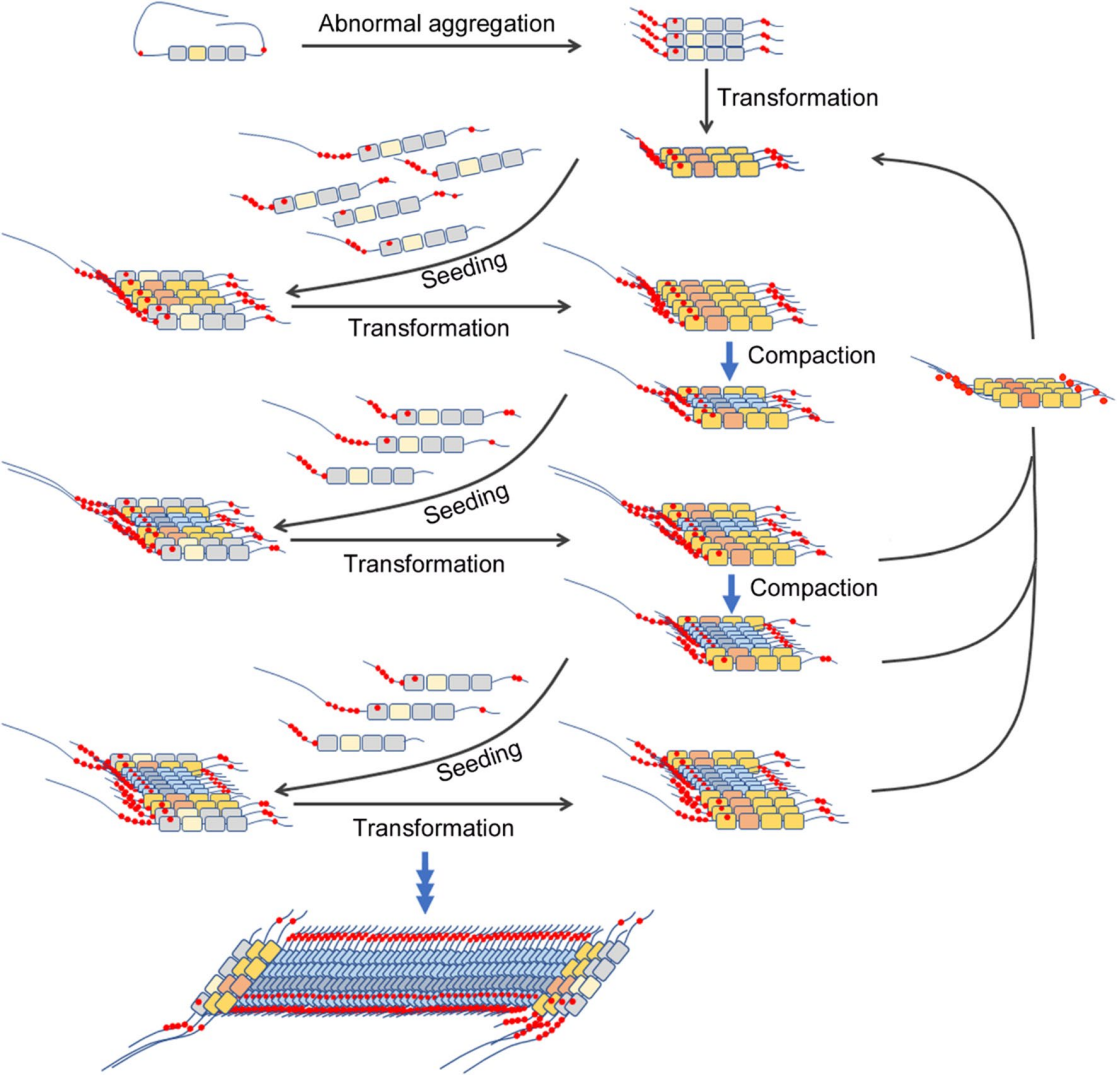
Proposed working model of the prion-like propagation of tau. Abnormally aggregated tau forms O-tau, which recruits tau and templates its transformation. Inner aggregates of tau are compacted to form compressed NFTs, SI_2_-tau, in which the prion-like activities are passivated. Outer aggregates of tau, SI_1_-tau, on one hand recruit normal tau and template the transformation, resulting in the growth of the NFTs. On the other hand, these aggregates may be released to form O-tau, which seeds to form new aggregation in the cells or in new cells

Taken together, we propose a working model of tau propagation (Fig. 9) in which O-tau recruits normal tau and templates the recruited tau and transforms to β-sheet conformation, resulting in loose aggregates, which recruit and template normal tau transformation. During the progression of tau pathogenesis, inner aggregated tau is truncated and condensed to form compressed aggregates, resulting in loss of its prion-like activities. Based on this model, we speculate that O-tau may initiate tau aggregation, but SI_1_-tau may contribute to the growth of aggregated tau.

## Data Availability

The datasets generated and/or analyzed during the present study are available from the corresponding author, Dr. Fei Liu, upon reasonable request.
